# Continuous professional competence (CPC) for emergency medical technicians in Ireland: educational needs assessment

**DOI:** 10.1186/1471-227X-13-25

**Published:** 2013-12-17

**Authors:** Shane Knox, Walter Cullen, Colum Dunne

**Affiliations:** 1 Centre for Interventions in Infection, Inflammation & Immunity (4i) and Graduate Entry Medical School, University of Limerick, Limerick, Ireland; 2 Health Services Executive, National Ambulance Service College, Dublin, Ireland

**Keywords:** Emergency medical technicians, Continuous professional development, CPD, Blended learning, E-learning, Educational, Ambulance

## Abstract

**Background:**

As in other countries, the Irish Regulator for Pre-Hospital practitioners, the Pre-Hospital Emergency Care Council (PHECC), will introduce a Continuous Professional Competence (CPC) framework for all Emergency Medical Technicians (EMTs), Paramedics and Advanced Paramedics (APs). This framework involves EMTs participating in regular and structured training to maintain professional competence and enable continuous professional developments. To inform the development of this framework, this study aimed to identify what EMTs consider the optimum educational outcomes and activity and their attitude towards CPC.

**Methods:**

All EMTs registered in Ireland (n = 925) were invited via email to complete an anonymous online survey. Survey questions were designed based on Continuous Professional Development (CPD) questionnaires used by other healthcare professions. Quantitative and qualitative analyses were performed.

**Results:**

Response rate was 43% (n = 399). 84% of participants had been registered in Ireland for less than 24 months, while 59% had been registered EMTs for more than one year. Outcomes were: evidence of CPC should be a condition for EMT registration in Ireland (95%), 78% believed that EMTs who do not maintain CPC should be denied the option to re-register. Although not required to do so at the time of survey, 69% maintained a professional portfolio and 24% had completed up to 20 hours of CPC activities in the prior 12 months. From a list of 22 proposed CPC activities, 97% stated that practical scenario-based exercises were most relevant to their role. E-learning curricula without practical components were considered irrelevant (32%), but the majority of participants (91%) welcomed access to e-learning when supplemented by related practical modules.

**Conclusion:**

EMTs are supportive of CPC as a key part of their professional development and registration. Blended learning, which involves clinical and practical skills and e-learning, is the optimum approach.

## Background

Pre-hospital care in Ireland is provided by the Health Service Executive’s (HSE) National Ambulance Service (NAS) and (in parts of Dublin city) the ‘Dublin Fire Brigade’. Staff who respond to pre-hospital incidents are all trained to Paramedic or Advanced Paramedic (AP) level. In addition, pre-hospital care is provided at sporting and other public events by Emergency Medical Technicians (EMTs), mostly within the voluntary organisations: Civil Defence, Order of Malta Ireland, St. John Ambulance and the Irish Red Cross. All of these practitioners are registered with the regulating authority, Ireland’s Pre-Hospital Emergency Care Council (PHECC) [1].

Currently, once registered as a practitioner with PHECC there is no requirement to show evidence of competence, other than annual certification in Cardiopulmonary Resuscitation (CPR). In order to re-register practitioners must also complete a self-declaration form stating that they are currently practicing, are of good character and in good health and will commit to the PHECC Code of Conduct and Ethics. There is no current requirement to show evidence of any patient contacts, or to maintain a learning portfolio, or participate in skill maintenance programmes. PHECC licences are issued yearly, while re-registration occurs every three years.

In 1993, a report from the Irish government [2] stated that the ambulance service “forms a valued and integral part of the emergency services” and “was used as an extension of the hospital service with the objective of getting the patient into hospital as quickly as possible so that advanced medical treatment could be provided by a medical practitioner”, thus implying: 1) that advanced medical treatment could only commence within a hospital and 2) that the only purpose of the ambulance service was to provide transport for patients. The same report further recommended significant improvement in the quality of training provided to ambulance personnel. Reflecting its most recent iteration, this recommendation is furthered in the PHECC strategic plan (2011–2014) where the need to develop and implement a continuing professional competence (CPC) framework was stated [3]. However, translating advances in care guidelines into actual care delivered to patients poses many challenges associated with the effective acquisition of new knowledge and practical skills in addition to maintenance of existing expertise.

Previous studies have assessed Paramedic and Advanced Paramedic training and continuing education in Ireland [4-7]. However, in this study, we wished to determine, for the first time, the attitudes of EMTs in Ireland towards CPC, their suggested outcomes / preferred delivery format and relevance to their roles. We devised a short answer survey, based on similar questionnaires used by other professions [8-12], to determine current EMT demographics, CPC activities, and attitudes towards effectiveness of the varying training methods employed. It is hoped that this information will inform future CPC programme development.

## Methods

### Participants

In July and August 2012, all EMTs licensed to practice in Ireland and registered with the Pre-hospital Emergency Care Council’s (PHECC) (n = 925) were contacted by email. Questions were entered into a Survey Monkey™ online questionnaire (http://www.surveymonkey.com). A link was provided to the survey and to a concise, unbiased explanation of the survey topic. Participation was voluntary and anonymous. Consent to participate was recorded. Conduction of the study and its design, taking into consideration published healthcare professions’ questionnaires relating to continuous professional development (CPD) [9-11,13], were approved by the Ethics Committee of the Faculty of Education and Health Sciences, University of Limerick, Ireland and the Research Ethics Committee of the Health Services Executive Mid-Western Regional Hospital, Limerick, Ireland.

### Data collection and analysis

Health professionals are increasingly expected to identify their own learning needs through self-assessment [14,15]. Therefore, the survey questions were designed to elicit participants’ views on CPC and, so, the survey was piloted following a presentation on CPC to 120 registered EMTs at a biannual conference in 2011 [16]. Responses were recorded (included audio recording) and summarised at the event using mind mapping software [17]. Following analysis of the exercise, the design of the questionnaire was finalised and trialled using 12 EMTs who were subsequently excluded from the analyses.

The questionnaire (see Additional file 1) comprised questions relating to demographics, opinions on CPC, registration and also included a matrix of 22 listed activities whereby participants were asked to indicate how relevant they believed each activity was to CPC. Some of the activities related to education generally, while others related specifically to pre-hospital practice. There were 26 items in the questionnaire. Not every single question was answered by every respondent and, therefore, answers are described by number and percentage of responses to specific questions. The data were downloaded from Survey Monkey™ software to an electronic data file and quantitative analysis was performed using Statistical Packages for the Social Sciences (SPSS version 20.0).

## Results

### Demographics

399/925 responses were received (43% of all registered EMTs), of whom 271 (68%) were Male; 115 (29%) were Female and 13 (3%) did not report gender. Table 1 compares the Age category with Gender.

**Table 1:** Gender and age group

			**Please select your appropriate age group**					**Total**	**All EMTs with emails registered with PHECC (N = 925)**
			**18- 21**	**22-29**	**30-39**	**40-49**	**Over 50**		
Gender	Male	Count	9	61	82	64	55	271	
		% within age group	69.2%	66.3%	70.1%	77.1%	67.9%	68%	
		% of total	2.3%	15.8%	21.2%	16.6%	14.2%	68%	69% (634)
	Female	Count	4	31	35	19	26	115	
		% within age group	30.8%	33.7%	29.9%	22.9%	32.1%	29%	
		% of total	1.0%	8.0%	9.1%	4.9%	6.7%	29%	31% (291)
Total		Count	13	92	117	83	81	386 (DNRG*)13	
		% of total	3.4%	23.8%	30.3%	21.5%	21.0%	100.0%	100% (925)

DNRG* – Did not report gender.

**Figure 1:**
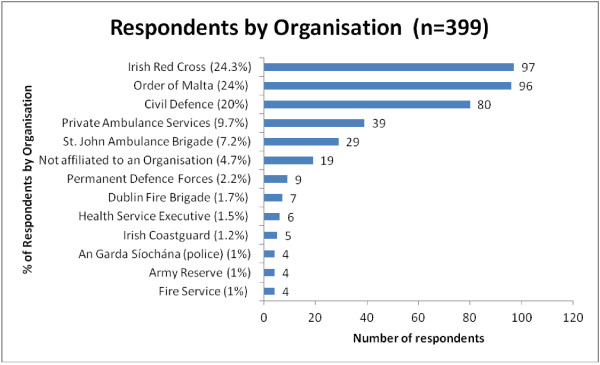
Respondents by organisation.

However, while responses were reasonably well dispersed (Figure 1) across the voluntary organisations: i.e., Order of Malta (96, 24%), Civil Defence (80, 20%), St. John Ambulance Brigade (29, 7%) and the Irish Red Cross (97, 24%), there was considerably less participation by EMTs employed by the Irish State (10%) such as the Permanent Defence Forces, Irish Health Service, An Garda Síochána (Police), etc and private ambulance services 9.7%. It should be noted that there were very few EMTs within these organisations at the time of the survey (as they are not employed in their permanent position as EMTs but may have completed the programme independently).

A total of 325 (84%) of respondents were registered EMTs for two years or less (Table 2), with almost half of those (161) being registered for less than one year. Respondents who had been with their organisation for less than five years represented 33% (n = 131) of the total surveyed, while 28% (n = 113) of those with less than five years service within their organisation had been registered as EMTs for less than two years. 21% of respondents had over 20 years experience with their respective organisations while 34% had less than six years service. 30–39 year old respondents represented 30% (n = 118) of the total responses and also represented the largest age group of those with their Organisation for less than five years.

**Table 2:** Participants’ length of service and registration with regulatory authority

			**Registration with the pre-hospital emergency care council (PHECC)**					**Total**
			**Up to 1 year**	**1 - 2 years**	**3 - 4 years**	**5 - 6 years**	**More than 6 years**	
Years with current organisation	0-5	Count	68	45	17	1	0	131
		% of total	17.6%	11.7%	4.4%	.3%	.0%	33.9%
	6-10	Count	43	28	16	1	0	88
		% of total	11.1%	7.3%	4.1%	.3%	.0%	22.8%
	11-15	Count	22	34	4	1	0	61
		% of total	5.7%	8.8%	1.0%	.3%	.0%	15.8%
	16-20	Count	8	16	2	0	0	26
		% of total	2.1%	4.1%	.5%	.0%	.0%	6.7%
	over 20	Count	20	41	13	0	6	80
		% of total	5.2%	10.6%	3.4%	.0%	1.6%	20.7%
Total		Count	161	164	52	3	6	386
		% of total	41.7%	42.5%	13.5%	.8%	1.6%	100.0%

### Attitudes towards continuous professional competence

CPC is considered extremely important by 86% (n = 343) of the EMTs surveyed. 82% (n = 329) agreed that all EMTs should maintain evidence of CPC activities. A total of 61% (n = 243) agreed that CPC is the sole responsibility of the registered practitioner (strongly agreed 26%, n = 104 and agreed 35%, n = 139). Over 78% of respondents (n = 313) believed that their organisation should have input, at least to some extent, into what components should constitute an individual’s CPC, with only 7% (n = 26) stating that the organisation should not have input. Of the EMTs surveyed, (39%, n = 154) disagreed that only the regulatory body (PHECC) should determine the structure of CPC components, while 26% (n = 105) agreed that only the PHECC should determine the structure of CPC.

### Linking continuous professional competence activities and registration

The majority of EMTs surveyed (69%, 220/321), although not obligated, maintained a professional portfolio at the time of the survey (Table 3), with 24% (n = 97) stating that they had completed up to 20 hours of CPC over the previous 12-month period. 11% (n = 43) claimed that they had completed over 100 hours of CPC in the same period. Notably, almost a quarter (23%, n = 91) of those who had completed their CPC in the previous year had funded participation themselves, while 29% (n = 116) had their costs covered by their organisation either partially (12%, n = 46) or in full (18%, n = 70). When queried as to appropriate levels of CPC required, given a range of choices: 20 hours; 21–40 hours; 41–60 hours; 61–80 hours and 81–100 hours almost 40% (n = 159) believed that an EMT should complete 20–40 hours annually (a combination of the first two categories), with only 8% (n = 34) stating that 81–100 hours would be appropriate.

**Table 3:** Attitudes towards CPC and linking CPC activities and registration

**Attitudes towards continuous professional competence (CPC)**	**Agree**	**Number of responses**
CPC is extremely important to me	86%	343
EMTs should maintain evidence of CPC activities	82%	329
CPC is the sole responsibility of the registered practitioner	61%	243
Your organisation should have some input into your CPC	78%	313
Only PHECC should determine the structure of CPC	26%	105
Linking CPC activities and registration		
Currently maintain a professional portfolio	69%	220/321
How many hours of CPC have you completed over the previous 12- month period?		
Up to 20 hours	24%	97
Over 100 hours	11%	43
Who paid for your CPC over the previous 12-month period?		
Self-funded	23%	91
Paid for by your Organisation – in full	18%	70
Paid for by your Organisation – partially	12%	46
How many hours of CPC activities do you think would be appropriate for EMTs in a 12-month period?	Agree	Number of responses
20 hours	14%	58
21-40 hours	25%	101
41-60 hours	17%	69
61-80 hours	8%	31
81-100 hours	8%	34
Other	9%	37
Skipped question	17%	69
EMTs who do not maintain their CPC and who continue not to meet the requirements, should not be allowed to re-register as an EMT	78%	273/352
Evidence of CPC should be a condition for EMT registration	95%	341/359
Registration as an EMT with PHECC is of personal importance	95%	381

Over 78% (273/352) of the EMTs surveyed stated that EMTs who do not maintain their CPC and continue not to meet the requirements, should not be allowed to re-register. 95% of respondents either strongly agreed (61%, 218/359), or agreed (34%, 123/359), that evidence of CPC should be a condition for EMT registration. 95% (n = 381) stated that registration with PHECC was of personal importance to them.

### Consultation regarding specific continuous professional competence activities

Most respondents considered practical type learning relevant (Table 4): training on a simulation manikin 92% (297/321), regular practical assessments 79% (253/319); Cardiac First Response (CFR/CPR) re-validation 97% (311/322); practical training scenarios 97% (313/321); completing a duty with paramedics/advanced paramedics 95% (306/321) and Annual Major Incident exercises 92% (297/319).

**Table 4:** Relevance of potential CPC activities

**Very relevant/relevant = relevant not relevant/very irrelevant = not relevant**	**Relevant**		**Not relevant**		
	**Responses**	**% of total responses**	**Responses**	**% of total responses**	**Total response to question**
Practical training scenarios	313	97%	2	0.6%	321
Annual cardiac first response/CPR revalidation	311	97%	6	2%	322
Attending courses accredited by PHECC	307	96%	2	0.6%	319
Doing a duty with paramedics/advanced paramedics	306	95%	7	2%	321
Major Incident/emergency exercises	297	93%	7	2%	319
Training on a simulation manikin	297	92%	7	2%	321
Access to e-learning followed by related practice	291	91%	5	2%	320
Keeping a portfolio of CPC activities	288	90%	4	1%	319
Mentoring others	277	87%	12	4%	317
Lecturing/teaching	276	86%	15	5%	319
Access to medical journals/medical books	266	83%	11	3%	320
Regular practical assessments	253	79%	13	4%	319
Being a tutor	251	79%	19	6%	316
Appraisal with senior EMT Officer (or above)	248	78%	20	6%	319
Relevant conferences e.g RESUS	246	78%	18	6%	317
Being an examiner	222	69%	30	9%	319
Appraisal with a doctor/medical supervisor	207	65%	37	11%	320
Case study review	204	64%	20	6%	317
First aid competitions	159	50%	78	25%	315
Project work	152	48%	50	16%	318
Appraisal of journal publications	124	39%	62	20%	316
e-learning modules only and no related practice	109	35%	101	32%	313

With regard to access to e-learning followed by related practice: 91% of respondents (291/320) believed this to be very relevant (45%, n = 145) or relevant 46% (n = 146); compared with ‘e-learning modules only and no related practice being very relevant 9% (n = 29) and relevant 26% (n = 80). ‘E-Learning modules only and no related practice’ recorded the highest ‘Very Irrelevant’ (8%, 27/313)/“irrelevant’ 24% (74/313) responses from all categories with a combined total of 32% (101/313) claiming it has no relevance.

In addition to the practical-type, hands-on activities preferred for CPC maintenance, EMTs also considered the following activities very relevant or relevant in maintaining Continuous Professional Competence: courses accredited by PHECC 96% (307/319); keeping a learning portfolio 90% (288/319); mentoring others 87% (277/317); lecturing/teaching 86% (276/319); being a Tutor 79% (251/316); attending relevant conferences 78% (246/317); appraisal with a senior EMT officer (or above) 78% (248/319); case study review 64% (204/317); being an examiner 69% (222/319); appraisal with a doctor/medical supervisor 65% (207/320); first aid competitions 50% (159/315); project work 48% (152/318); appraisal of a journal publication 39% (124/316).

## Discussion

Whilst there is evidence of competence and CPD programmes within ambulance services internationally (e.g., Norway [18], Australia [19], UK [20], Canada [21]), the evidence of any consultation with practitioners prior to the introduction of such programmes is scarce.

EMTs must embrace the multitude of activities that contribute to a professional’s development and the outcome of good CPD should be practitioners with increased competence and improved patient care [22]. This is the first study of attitudes towards professional competence among EMTs in Ireland and indicates that there appears to be a genuine enthusiasm for the introduction of CPC and a positive link to professionalism, similar to other healthcare professions [9,11,12,23-26]. This enthusiasm towards CPC is reinforced further as a significant number of EMTs are already maintaining a learning portfolio and participating in CPC activities, as the vast majority of participants agreed that CPC should be a requirement for PHECC registration and as 95% believed that registration with PHECC is of personal importance to them. This view of CPD being a requirement for registration is supported by legislation for some professions [27-29] or shown in previous studies to be shared by practitioners themselves [26,30].

### E-learning

E-learning is the use of internet technologies to enhance knowledge and performance [31]. There are many formats in which e-learning is delivered and many terms synonymous with e-learning, such as web-based (WBL) or on-line learning. One of the advantages of e-learning is that it can be synchronous or asynchronous and, therefore, can be flexible and particularly attractive for pre-hospital practitioners. In Ireland, PHECC has progressed the use of on-line examinations and learning modules since its formation. Indeed, Irish EMT examinations are delivered partially via an electronic software programme. Most Health Professions regulators tend to accredit and set standards in training rather than develop training [32,33] and, so, the e-learning approach (albeit blended with practical instruction provided by the training institutions) utilised most recently by PHECC to allow paramedics and APs complete on-line learning modules is unusual. Furthermore, this training methodology had not been in place for the initial training of EMTs surveyed and, taking cognisance of the survey results, it would appear that EMTs might use e-learning followed by practical reinforcement, but would appear less eager to use e-learning alone as a means to maintain competence.

Our survey included 22 potential CPC activities (see Table 4) and asked which activities did EMTs believe were relevant/irrelevant. The results showed that practical, hands-on activities were preferred over theoretical/non-practice type activities. Also, there were less negative responses regarding activities related to practical skills than to theoretical skills. This further substantiates the case for practical, hands-on activities, whether as a standalone activity or coupled with the e-learning approach. The EMTs surveyed in this study seemed to share the view of Ruiz *et al* in that perhaps they did not value e-learning as a replacement for traditional instructor-led training but rather as a complement to it, forming part of a blended-learning strategy [31]. EMTs function in environments that require lateral thinking [34]. Arguably, variation in learning methodologies could be encouraged so to facilitate the variations in personal learning styles while also taking cognisance of nuances in practice.

Previous studies with Irish advanced paramedics and paramedics reinforce the concept of practical-type learning as a preferred methodology and as an effective way of maintaining skills [7,35] and that skills practice is an integral part of maintaining competence [36]. Indeed, our results, in part, reinforce the focus of older/traditional basic training curricula for ambulance staff in the United Kingdom and Ireland, which for the most part, was skills-based [37]. This is quite different to results seen for other professions who tend to prefer attending conferences, lectures and reading of relevant journals [9,12], even though there is little evidence to suggest that attending conferences had any direct impact on improving professional practice [38].

### CPC annual hours

Internationally, there are similarities in the way in which CPC hours are recorded, most being based on an hours-related credit system, in which one hour of educational activity equates to one credit and the number of credit/hours required vary from between 50–100 per year [30]. Irish doctors now, under the Medical Practitioners Act [27] must meet professional competence requirements [39] and this currently is 50 hours per year. In that context, the respondents in this survey believe that it would not be unreasonable to expect EMTs to complete 20–40 hours annually.

#### ***Limitations***

The study had a number of strengths and weaknesses. The majority of respondents were male 70% (n = 272) in what is predominantly a male dominated profession in Ireland. At the time of the survey, there were 634 males registered with PHECC representing 69% of all EMTs registered (n = 925). Thus, the sample of participants in this study was similar to the proportion of male EMTs registered with PHECC.

The response to this survey was quite favourable, with a response rate of over 40%. This too is perhaps not surprising and may be due to the fact that the EMTs surveyed, for the most part, were affiliated with the voluntary organisations and, by association, are enthusiastic volunteers who self-nominated to progress to EMT programmes and subsequent examinations.

Notably, one group of EMTs may not have participated. These are EMTs not affiliated to any organisation and who most likely completed the EMT training programme independently.

While the response to the survey was quite favourable, we acknowledge some methodological considerations may limit generalisability. For instance, while we report data from 399 responses, this represented 43% of all registered EMTs. Our study was limited to those with valid email addresses on the PHECC register and clearly those for whom the subject area was a priority. Therefore, it is possible that our sample may not be representative of EMTs in general. Furthermore, the fact that a significant number of respondents represented a younger population (with over 27% under the age of thirty years, and a further 30% under the age of forty years) may have influenced the results. Arguably, a younger population may prefer a blended learning approach with an active participation and e-learning combination given the possibility that they may be more familiar with on-line/e-learning experiences. Indeed, the length of the survey may have been perceived as too long or complex, thereby reducing the return rate. Further research following the introduction of CPC for EMTs may expand upon these findings.

## Conclusions

To date, little research has been conducted with PHECC registered practitioners in general or on EMTs and CPD/C internationally. This survey is the first to ascertain the opinions of EMTs regarding CPC in terms of what is being completed currently, and how it may be developed in Ireland in the coming years.

The results of this survey demonstrate, at the very least, emphasis will need to be placed on practical activities such as: Cardiac First Response, maintaining a portfolio of evidence, mentoring others, completing operational shifts with paramedics and advanced paramedics and a blended learning approach with e-learning.

Conversely, less emphasis should be placed on e-learning alone and prudent purveyors of education for pre-hospital practitioners should emphasise inclusion of practical-type education.

There appears to be a genuine enthusiasm towards CPC, with a large number of EMTs already completing CPC activities, maintaining a learning portfolio and maintaining their registration. Maintaining this motivation is an important facet of effective professional competence and development.

## Competing interests

The authors declare that they have no competing interests.

## Authors’ contributions

SK conceived of the study and was involved in the design, collection of data, data analysis, drafting the manuscript. WC and CD (principal investigator) were involved in the conception of the study, data analysis and interpretation and drafting the manuscript. All authors read, reviewed the manuscript critically for intellectual content, and approved the final manuscript.

## Authors’ information

Prof Colum Dunne, Chair & Director of Research, Graduate Entry Medical School, University of Limerick, Limerick, Ireland. Tel: +353 (0)61 234703. Email: colum.dunne@ul.ie

SK: Education Manager, National Ambulance Service College, Dublin, Ireland.

WC: Chair of General Practice, Graduate Entry Medical School, University of Limerick, Ireland.

CD: Chair & Director of Research, Graduate Entry Medical School, University of Limerick, Ireland.

## Pre-publication history

The pre-publication history for this paper can be accessed here:

http://www.biomedcentral.com/1471-227X/13/25/prepub

## Supplementary Material

Additional file 1 Emergency Medical Technician Continuous Professional Competence questionnaire. Click here for file
