# Supplementary material for: Continuous professional competence (CPC) for emergency medical technicians in Ireland: educational needs assessment
Source: BMC Emerg Med. 2013 Dec 17;13:25. doi: 10.1186/1471-227X-13-25 (PMC3898252; doi:10.1186/1471-227X-13-25)
Supplement: Additional file 1 — Emergency Medical Technician Continuous Professional Competence questionnaire. [file 1471-227X-13-25-S1.pdf]

Additional File 1

Emergency Medical Technician – CPC Questionnaire

Please tick the boxes as appropriate in answer to the following questions

1. Are you currently registered as an EMT with PHECC?

   Yes No

2. How long have you been registered as an EMT?
   Up to 1 year 1 up to 2 years 2 to up to-3 years 3 to up to 5 years 5 years or more

3. Registration with PHECC as a pre-hospital practitioner is of personal importance to you.
   Strongly Agree Agree Undecided Disagree Strongly Disagree

4. Which Organisation are you a member (employee) of?
   Civil Defence Order of Malta St. John Ambulance Brigade Irish Red Cross
   Permanent Defence Forces Private Ambulance Service
   Other (please state name of organisation)

5. How many years have you been with this organisation?
   0-5 6-10 11-15 16-20 over 20

6. Please select your appropriate age group.
   18-21 22-26 27-30 31-35 36-40 41-45 46-50 over 50

7. Gender
   Male Female

The Term ‘Continuous Professional Competence ‘(CPC) refers to activities that contribute to your professional development by being able to demonstrate competency related to your EMT status.
PHECC intend to introduce CPC across all registration levels. The registered practitioner must show evidence of CPC to maintain their registration as an EMT. Please answer the following questions relating to CPC.

Please give your opinion on the statements were given

8. It is right that evidence of Continuous professional competence is a condition for PHECC registration as an EMT.
   Strongly Agree Agree Undecided Disagree Strongly Disagree

9. It is the sole responsibility of the registered practitioner to maintain their own CPC.
   Strongly Agree Agree Undecided Disagree Strongly Disagree

10. Should your organisation have input into what components should make-up an individual’s CPC?
   Yes To some extent No Not sure

11. Your CPC portfolio, or record of CPC evidence, should be available for audit by your Organisation.
   Strongly Agree Agree Undecided Disagree Strongly Disagree

12. Only PHECC should determine what components make-up and individual’s CPC.
   Strongly Agree Agree Undecided Disagree Strongly Disagree

13. PHECC, your organisation and the registered practitioner should decide on the activities that may be used for CPC.
   Strongly Agree Agree Undecided Disagree Strongly Disagree

14. CPC is extremely important to me as a registered pre-hospital practitioner.
   Strongly Agree Agree Undecided Disagree Strongly Disagree

15. To ensure registration, all EMTs should maintain evidence of CPC activities.
   Strongly Agree Agree Undecided Disagree Strongly Disagree

16. EMTs who do not maintain their continuous professional competence, and who continue not to meet the requirements, should not be allowed to re-register.
   Strongly Agree Agree Undecided Disagree Strongly Disagree

A professional portfolio is a personal collection of evidence which demonstrates the continuing acquisition of skills, knowledge, attitudes, understanding and achievements.

17. Do you maintain a professional portfolio?
    Yes No

18. Please indicate the number of CPC hours you have recorded over the last 12 months
    None < 20 hrs 21-40 hrs 41-60hrs 61-80hrs 81-100hrs >100hrs

19. If you have completed CPC activities in the previous 12 month period who funded it?

    Not completed CPC Self funded Funded by Organisation

    Partially funded by Organisation CPC activities provided Free of charge

20. How many hours of CPC activities do you think would be appropriate in a 12 month period for EMTs? Please give ranges here.

21. Please indicate how relevant you believe the activities listed below are to you maintaining your Continuous Professional Competence.

(for all) Very relevant relevant Undecided not relevant very irrelevant

- Training on a simulation manikin
- Relevant Conferences e.g RESUS
- Regular practical assessments
- Project work
- Practical Training scenarios
- Mentoring others
- Major Incident/Emergency exercises
- Lecturing/teaching
- Keeping a portfolio of CPC activities
- First Aid Competitions
- e-learning modules only and no related practice
- Doing a duty with paramedics/advanced paramedics
- Case Study review
- Being an examiner
- Being a Tutor
- Attending courses accredited by PHECC
- Appraisal with senior EMT Officer (or above)
- Appraisal with a Doctor/medical supervisor
- Appraisal of journal publications
- Annual Cardiac First Response/CPR revalidation
- Access to medical journals/medical books
- Access to e-learning followed by related practice

22. Your Organisation only should decide what activities you must do for CPC each year.

Strongly Agree Agree Undecided Disagree Strongly Disagree

23. You and your Organisation should have input into what CPC activities are required each year.

Strongly Agree Agree Undecided Disagree Strongly Disagree

24. Your organisation should have no input at all into deciding what constitutes CPC.

Strongly Agree Agree Undecided Disagree Strongly Disagree

25. PHECC should require a minimum of standard CPC activities, such as completed patient care reports, evidence of cardiac first response revalidation, evidence of managing patients, but other activities should be allowed if considered relevant by the practitioner.

Strongly Agree Agree Undecided Disagree Strongly Disagree
